# Cross-cultural adaptation and cognitive interview-based content validation of the English Participation Behaviour Questionnaire (PBQ), to measure participation in individuals with hand injuries

**DOI:** 10.1177/17589983251403595

**Published:** 2025-11-26

**Authors:** Maryam Farzad, Rochelle Furtado, Joy MacDermid

**Affiliations:** 1 Occupational Therapy, Hand and Upper Limb Center, St. Joseph’s Health Center, School of Physical Therapy, Department of Health and Rehabilitation Sciences, 6221 Western University, London, ON, Canada; 2 48533 University of Social Welfare and Rehabilitation Sciences, Tehran, Iran; 3 University Health Network, 697514 KITE Research Institute, Toronto, ON, Canada; 4 Physical Therapy and Surgery, 6221 Western University, London, ON, Canada; 5 Clinical Research Lab, Hand and Upper Limb Center, St. Joseph’s Health Center, London, ON, Canada; 6 Rehabilitation Science, 62703 McMaster University, Hamilton, ON, Canada

**Keywords:** hand injuries, participation, content validity, cross-cultural adaptation, patient-reported outcomes, cognitive interviewing

## Abstract

**Background:**

Hand injuries can significantly impair individuals’ ability to engage in essential daily and social activities, necessitating valid and culturally relevant tools to assess participation limitations. The Participation Behaviour Questionnaire (PBQ), originally developed in Persian and grounded in the International Classification of Functioning, Disability and Health (ICF), was designed to evaluate the extent of participation restrictions following hand and upper limb injuries.

**Purpose:**

This study aimed to translate and cross-culturally adapt the PBQ for use among Canadian English-speaking individuals with hand injuries and assess its content validity within a Canadian clinical context.

**Methods:**

The PBQ was adapted according to Beaton’s five-step guideline for the cross-cultural translation of self-report measures. Cognitive interviews were conducted with 15 patients and 22 healthcare professionals, including physiotherapists, hand therapists, surgeons, and rehabilitation researchers, using think-aloud and semi-structured methods to examine item clarity, consistency of interpretation, and cultural appropriateness.

**Findings:**

Cognitive interviews with 15 patients and 22 experts revealed 25 items requiring revision, with 88% of concerns related to clarity and comprehension. Overall, clarity issues were identified in 22 of the 37 items, resulting in 18 substantive revisions. For example, the item “I feel I have lost my autonomy” was revised to “I feel I have lost my independence in daily tasks” to improve clarity. Commonly misunderstood terms included “public transport” and “voluntary job,” which were refined using culturally contextual examples. Cultural and contextual factors also influenced how participants interpreted items such as “My use of public transport” (12%) and “Engagement in voluntary work” (20%).

**Implications:**

The English-adapted PBQ demonstrated evidence of content validity based on participant feedback regarding clarity, relevance, and comprehensibility. The 18 substantive revisions enhanced cultural and linguistic appropriateness by addressing clarity in 22 of 37 items, refining ambiguous terminology, and incorporating Canadian-contextual examples where necessary. These findings represent an initial step in the overall validation process; in this pre-psychometric, single-center Canadian-English study, additional research is required to assess the instrument’s psychometric properties, including construct validity, reliability, and responsiveness.

## Introduction

Hand injuries are among the most debilitating conditions, significantly impacting individuals’ ability to perform essential daily activities, roles, and occupations such as self-care, work, and leisure, ultimately affecting their quality of life and psychosocial well-being.^1,2^ Participation has therefore been increasingly recognized as a critical outcome in hand therapy, since these injuries disrupt engagement in meaningful roles such as self-care, work, family responsibilities, and leisure^3,4^ While traditional biomechanical approaches focus on restoring strength, mobility, and dexterity, these measures alone do not capture the broader impact of hand impairment on a person’s social functioning, autonomy, and life satisfaction.^5^

*Despite the importance of participation, measuring it remains challenging in hand therapy.* Upper extremity injuries can profoundly disrupt individuals’ ability to resume key life roles, including employment, caregiving, and social engagement.^5–8^ Traditional functional assessments typically focus on impairments (e.g., range of motion, grip strength) or activity limitations (e.g., buttoning a shirt), but do not capture the broader impact of injury on an individual’s social identity, autonomy, or role fulfillment.^9^ Participation refers to involvement in real-life situations, such as working, parenting, or engaging in leisure activities, and is shaped by both personal and environmental factors. Evaluating participation offers insight into how hand conditions impact everyday functioning beyond physical outcomes, particularly when patients appear functionally recovered but continue to be restricted in meaningful roles.

Although several generic instruments assess participation, they often lack relevance for upper limb conditions and may not capture the nuanced impact of hand impairment on valued occupations.^10,11^ In contrast, participation-focused tools offer clinically relevant information to guide rehabilitation, promote client-centred care, and support engagement in daily life.^3,12,13^

The International Classification of Functioning, Disability, and Health (ICF) provides a comprehensive framework for understanding participation as “involvement in life situations.”^14^ Within this model, participation is shaped not only by physical impairments but also by personal and environmental factors that influence an individual’s ability to engage in meaningful roles. In the context of hand injuries, the ICF emphasizes that even localized impairments can lead to significant disruptions in work, self-care, and social participation.^15–17^ By emphasizing dynamic interactions between individuals and their environments, the ICF supports the development of outcome measures that move beyond impairment-level metrics and capture participation-centred rehabilitation needs.^18–20^

The ICF framework emphasizes the importance of outcome measures that encompass the comprehensive scope of recovery, extending beyond physical healing to include the restoration of social roles, autonomy, and participation in life. However, few condition-specific tools have been developed to assess participation among individuals with hand injuries.^3,18,21^

The Participation Behaviour Questionnaire (PBQ) was developed as a theoretically grounded and psychometrically robust instrument to assess participation limitations specifically in individuals with hand and upper extremity injuries.^22^ Its development was informed by the International Classification of Functioning, Disability and Health (ICF) framework, with a focus on participation as involvement in life situations and emphasis on the influence of environmental and personal factors on engagement. In parallel, the PBQ integrates principles from contemporary occupational therapy models, including the Person-Environment-Occupation-Performance (PEOP) model and the Canadian Model of Occupational Performance and Engagement (CMOP-E), both of which extend beyond task performance to consider satisfaction, autonomy, and volitional engagement in meaningful roles. These complementary frameworks ensure that the PBQ does not narrowly measure activity limitations but captures a multidimensional view of participation that includes perceived role fulfillment, interpersonal connectedness, and leisure engagement. The PBQ’s four content domains—Social Participation & Interpersonal Relationships, Autonomy & Role, Subjective Satisfaction with Participation, and Recreational, Sport & Leisure Time—reflect this conceptual depth and offer clinicians a comprehensive tool to assess the broader impacts of hand conditions on daily life. By linking ICF concepts with occupational therapy perspectives, the PBQ is uniquely positioned to inform both clinical reasoning and outcome measurement in participation-centred rehabilitation.

Although the PBQ demonstrated strong psychometric properties in its original Persian-language context, its direct use in English-speaking clinical settings is limited by differences in language, social norms, and healthcare delivery systems.^23,24^ Participation is a culturally sensitive construct shaped by environmental, societal, and linguistic factors that influence how individuals engage in roles and activities.^25,26^ As such, valid cross-cultural adaptation requires more than direct translation—it demands careful attention to preserving the instrument’s conceptual integrity. Established frameworks such as Beaton’s guidelines and the COSMIN methodology recommend a rigorous, multi-step process that includes both linguistic translation and evaluation of content validity to ensure that items remain meaningful and relevant in the target context.^24,27,28^ Reverse adaptation, from non-English into English, is less common but equally important, as it preserves the conceptual richness developed outside the Anglophone context and aligns it with Canadian clinical practice. Without careful adaptation, conceptual mismatches may introduce measurement bias and reduce clinical utility.

This study, therefore, aimed to conduct a systematic cross-cultural adaptation and content validation of the PBQ for use in Canadian English-speaking populations, ensuring that items retained conceptual fidelity while being culturally and linguistically appropriate.

## Methods

### The Participation Behaviour Questionnaire (PBQ)

The Participation Behaviour Questionnaire (PBQ) is a 30-item self-report instrument developed in Persian to evaluate participation restrictions among individuals with hand and upper extremity injuries.^22^ The PBQ was developed using Item Response Theory (IRT) and Rasch modeling,^29^ ensuring a rigorous psychometric foundation. Cronbach’s alpha (0.96) and the Item Reliability Index (0.91) confirmed strong construct validity and measurement precision.^31^ In Rasch modelling, Cronbach’s alpha reflected the instrument’s unidimensionality and precision rather than traditional measures of internal consistency or test-retest stability.^30^

The PBQ is conceptually organized into four content-based subdomains that represent key dimensions of participation affected by upper limb impairments: (1) *Social Participation and Interpersonal Relationships* – assessing disruptions in social interactions and connectedness, (2) *Autonomy and Role* – evaluating limitations in fulfilling daily, occupational, and familial responsibilities, (3) *Subjective Satisfaction with Participation* – capturing perceived adequacy and satisfaction with current levels of engagement, and (4) *Recreational, Sport, and Leisure Time* – identifying limitations in non-obligatory, enjoyable activities.^31^

Each item refers to experiences over the past 2 weeks and is scored on a four-point Likert scale ranging from 0 (“totally disagree”) to 3 (“strongly agree”), with higher scores indicating greater participation restriction. Total scores range from 0 to 90.

Although Rasch analysis supports the PBQ’s unidimensional measurement structure—meaning it can be interpreted as a single overall construct—the four content domains may also be analyzed as subscales to provide more detailed insights into specific areas of participation restriction.

### Translation process

The translation and cross-cultural adaptation process followed established guidelines for self-report measures.^32^

1. Forward translation: Two bilingual translators whose native language was Persian with professional fluency in Canadian English one a hand therapist and one a professional translator) independently translated the questionnaire from Persian into English. The hand therapist ensured clinical relevance, while the translator provided a linguistic perspective.
2. Synthesis: A panel consisting of both translators and hand therapists compared and synthesized the two versions into a single reconciled English draft. Discrepancies were resolved during this stage to produce a single reconciled version. Some wording accepted at this stage was later reconsidered during cognitive interviews, highlighting differences between initial synthesis decisions and findings from qualitative analysis.
3. Backward translation: Two independent bilingual translators, both native Persian speakers with no medical background and blinded to the original Persian questionnaire, back-translated the reconciled English version into Persian. This ensured that the English version retained conceptual equivalence with the source.
4. Expert Committee Review: An expert committee, including translators, clinicians, and the original developer, compared the English version with the Persian source, focusing on semantic, idiomatic, experiential, and conceptual equivalence.^32^ Written documentation was maintained, and final decisions were made by consensus.
5. Content Validation: Qualitative methods, including cognitive interviews and the think-aloud approach, were employed with patients and occupational therapists to evaluate item clarity, cultural relevance, and interpretability. Data collection was conducted in two rounds at the Hand and Upper Limb Centre in London, Ontario Canada, during the summers of 2018 and 2023, ensuring appropriateness for a Canadian English–speaking context.

### Participants

We used purposeful sampling to recruit experts and patients, ensuring diverse perspectives and in-depth insights relevant to the research objectives.^33^ Participants were selected using purposive sampling to represent diversity in terms of age, gender, clinical diagnosis, and professional experience. While this approach supported variation in clinical perspectives, we acknowledge that cultural or religious diversity may not have been fully represented. We continued recruitment and data collection until content or code saturation was achieved, defined as the point at which no new issues emerged regarding item clarity, interpretation, or relevance. This decision was guided by established recommendations for cognitive interviewing, which support sample sizes of 10 to 15 participants and emphasize the adequacy of saturation over quantity.^34,35^

#### Expert participants

A total of 22 individuals were recruited as expert participants. Inclusion criteria required participants to have a minimum of 2 years of clinical experience in rehabilitation, with additional consideration given to academic training or research in participation-focused interventions. The expert group included clinicians and rehabilitation researchers with diverse professional backgrounds: three occupational therapists, two certified hand therapists, two physiotherapists, two hand surgeons, one orthopedic surgeon, and twelve PhD students in rehabilitation sciences, all of whom were licensed clinicians with prior professional experience in occupational or physical therapy and were undertaking doctoral training to further specialize in rehabilitation research. The inclusion of PhD students was intended to capture both advanced clinical expertise and research-oriented perspectives, thereby broadening the scope of feedback on participation constructs. This blend of professional expertise ensured representation of both practical and theoretical perspectives on participation. For transparency, we analyzed patient and expert perspectives separately. Within the expert group, feedback from PhD trainees and practicing clinicians was compared narratively rather than statistically weighted. Experts contributed qualitative feedback on the PBQ through cognitive interviews but were not involved in the final decision-making regarding item revisions. All item modifications were determined by the core research team, which included senior researchers, postdoctoral fellows, and graduate trainees. To mitigate potential power imbalances during analysis and consensus-building, junior team members were encouraged to share their interpretations first, and all decisions were reached collaboratively. A decision log was maintained to support auditability and transparency in the adaptation process.

#### Patient participants

Patients were recruited from a tertiary hand and upper limb rehabilitation clinic. Inclusion criteria required patients to be aged 18 years or older, diagnosed with a hand or upper limb condition, and cognitively and physically able to participate in interviews. Patients with comorbid mental health or neurological conditions that could impair participation in the study were excluded. In total, 15 patients participated in the cognitive interviews. The sample was predominantly female (70%), with an average age of 45 years, and represented a range of diagnoses related to traumatic or non-traumatic hand injuries (see Table 1).

**Table 1.** Participants’ demographic information.

Demographic	Patients (*n* = 15)	Experts (*n* = 22)
Age, mean (SD)	45 (6.5)	37 (6.5)
Sex		
Female	11 (70%)	9 (40%)
Male	4 (30%)	13 (60%)
Injured hand		
Right	10 (67%)	—
Left	5 (33%)	—
Dominant hand		
Right	12 (80%)	—
Left	3 (20%)	—
Diagnosis		
Wrist fracture	6 (40%)	—
Hand stiffness	3 (20%)	—
Shoulder replacement	2 (13%)	—
Osteoarthritis (OA)	4 (27%)	—
Occupation		
Manual	5 (33%)	—
Not manual	5 (33%)	—
Unemployed	3 (20%)	—
Homemaker	2 (13%)	—
Experience in treating patients (Years)	—	10 (3.5)
Profession		
Occupational therapists (OT)	—	3 (14%)
Certified hand therapists	—	2 (9%)
Hand surgeons	—	2 (9%)
Orthopedic surgeons	—	1 (5%)
Physiotherapists (PT)	—	2 (9%)
Health sciences students (OT and PT)	—	12 (54%)

#### Ethical considerations

This study received ethical approval from the University of Western Ontario Research Ethics Board (Approval Number: 114374). All procedures adhered to institutional and international ethical standards, including the Declaration of Helsinki. Written informed consent was obtained from all participants. To protect confidentiality, all transcripts were anonymized prior to analysis, and identifying information was removed from reporting.

### Data collection

RF (a female graduate student and physical therapist with 5 years of qualitative experience) and MF (a female postdoctoral fellow and hand therapist with over 5 years of qualitative experience) conducted the interviews, which lasted between 20 and 30 min. While most interviews took place face-to-face, public health social distancing guidelines required some sessions to be conducted via phone or online meetings. The authors developed the interview guide using cognitive interviewing methodology,^36^ and structured it based on the ICF to ensure the PBQ adaptation captured environmental, personal, and activity-based influences on participation. These models guided the alignment with occupation-centred practice, emphasizing participation as both a process and outcome in occupational therapy. (Appendix 1) Questions focused on how participants interpreted each PBQ item. During the interviews, a think-out-loud technique was employed, encouraging participants to share all their thoughts as they answered each item of PBQ.

Additionally, tailored open-ended questions and specific probes were used to delve deeper into the participants’ reasoning behind their responses. Questions like “Can you define this word?” or “Can you give an example?” helped explore the thought process behind each response. Participants also explained the rationale behind their answers. Discrepancies during both translation synthesis and qualitative analysis were resolved through consensus discussions between research team members. Interview sessions were audio-recorded and transcribed, and coding conflicts were resolved jointly by the two researchers (RF and MF). A decision log was maintained to document the rationale for revisions, supporting the auditability of the adaptation process. These cognitive interview sessions were recorded and transcribed for analysis by two researchers. Interview recruitment ceased when response saturation was achieved, identified when three consecutive interviews yielded no new information. Thus, each interview transcript was promptly analyzed to decide the necessity of further interviews.^37^

### Analysis

Interview data were analyzed using a structured coding framework to identify issues of clarity, comprehensibility, and relevance. A deductive approach was employed, utilizing an existing coding scheme to categorize and identify interpretation issues for each item. This previously established coding system was instrumental in classifying issues impacting item interpretation into six categories: Comprehension/Clarity (C), Perspective Modifiers (PM), Reference Point (RP), Calibration Across Items (CAI), Inadequate Response Definition (IR), and Relevance (R). This approach was chosen to align with COSMIN methodology, as the goal was to systematically evaluate whether the English-adapted PBQ met established content validity criteria. An inductive approach was not applied because the aim was not to generate new categories or theory, but to test the adapted PBQ against established domains of content validity.^38,39^ The deductive framework, therefore, ensured consistency, transparency, and comparability across items and participants while maintaining alignment with international methodological standards.

## Results

### Translation and cross-cultural adaptation

The cross-cultural adaptation of the Participation Behaviour Questionnaire (PBQ) was conducted in accordance with Beaton’s five-step guideline^32^, which emphasizes methodological rigour to ensure both linguistic and conceptual equivalence between the source and target versions.

#### Stage 1: Forward translation

Two independent translators, both fluent in English and native Persian speakers with professional backgrounds, independently translated the original PBQ into Persian. One translator was familiar with the concepts underlying the questionnaire, while the other was naïve to avoid bias. This dual approach helped uncover divergent interpretations of key terms. For instance, the term “public places” posed ambiguity—one translator rendered it as “general environments,” while the other used “open spaces.” The discrepancies highlighted subtle shifts in contextual meaning, especially about community participation.

#### Stage 2: Synthesis of translations

The two forward translations were synthesized into a single reconciled English version through a consensus meeting led by the translation coordinator. During this synthesis, considerable attention was given to items that were either culturally specific or involved complex constructs such as “voluntary job,” “family roles,” and “trophic changes.” To maintain conceptual integrity, the team prioritized culturally relevant yet semantically equivalent terms. For example, “social activities” was initially translated as “group activities.” However, after discussion, it was agreed that the phrase retained the broader notion of interpersonal engagement central to the original intent.

#### Stage 3: Back translation

Two separate bilingual translators, blinded to the original version and not involved in prior stages, independently backtranslated the synthesized English version into Persian. This step served as a quality check to identify any semantic drift or conceptual inconsistencies. The back-translated phrase “public areas” for “public places” was deemed an acceptable match. However, terms like “autonomy” and “voluntary job” revealed nuanced challenges, as their equivalents were interpreted differently across professional and cultural lines. The back translation of “voluntary job” as “group contribution” prompted a discussion on ensuring the term captured the full spectrum of unpaid work, including caregiving and community service.

#### Stage 4: Expert committee review

An expert committee composed of occupational therapists, certified hand therapists, physiotherapists, hand surgeons, and doctoral students in rehabilitation sciences convened to review all versions and reconcile any remaining discrepancies. This multidisciplinary team provided feedback not only on linguistic precision but also on clinical relevance and cross-cultural applicability. The committee flagged several items for clarification. For example, Item 6 (“I cannot use public transportation”) was questioned for potential misinterpretation, especially for rural or suburban populations. Following these discussions, revisions were made to several items to enhance clarity and ensure the retention of functional and contextual meaning. Calibration issues in response options were also flagged, and a plan for further validation in the pre-testing phase was developed.

#### Stage 5: Pre-testing through cognitive interviews

Pre-testing was carried out using structured cognitive interviews with 37 participants—22 experts and 15 patients. These interviews provided insight into how real users interpreted the adapted PBQ. Terminology comprehension, cultural relevance, and item clarity were systematically evaluated. Multiple participants raised concerns about vague or technical terms. For instance, a patient (P12) commented on Item 23:

“What kind of changes do you mean? Skin colour? Temperature? This needs explanation. I had no idea what that word meant.”

Similarly, a participant (P04) described the emotional nuance behind social participation:

“It is not that I avoid people—I just do not want them to see my hand like this. That is different from not wanting to go.”

Issues were coded using a standardized framework, and the research team identified 25 problematic items requiring revisions, most commonly due to clarity (22 items), reference point inconsistencies (4 items), and limited relevance (1 item). These findings were discussed in regular team meetings and documented in a decision log to ensure transparency and traceability.

Although the original developer was consulted during the early stages of translation, final decisions regarding item revisions were discussed and agreed upon by the expert committee to support a collaborative and multidisciplinary approach. Efforts were made to identify and address discrepancies between the original Persian and back-translated English versions, in line with Beaton’s guidelines.

### Content validation through cognitive interviews

A total of 37 individuals, 22 expert participants and 15 patients were engaged in the cognitive interviewing phase. This phase was designed to assess how respondents understood, interpreted, and responded to each item in the English version of the PBQ, with a specific focus on the tool’s relevance, clarity, and comprehensiveness in a Canadian rehabilitation context.

#### Experts’ perspective

Experts flagged 20 items (54%) as problematic; 16 overlapped with patient concerns, and four were unique to the expert group. Most issues fell under the COSMIN domain of comprehensibility, with 18 of 37 items (49%) judged unclear or context dependent. A recurring issue was the clarity of wording. For example, Item 3 (“I can participate in family activities”) generated divergent interpretations:

“Are we talking about holiday dinners or daily care routines? Family roles are so culturally loaded; we need to define them.” (E03)

Similarly, Item 6 (“I cannot use public transportation”) raised concerns about conflating functional capacity with service availability:

“Many of my rural patients simply do not have access to public transport—it is not about their functional capacity.” (E14)

Other terminology was also problematic. The phrase “voluntary job” was interpreted inconsistently:

“Does this mean community volunteering, or informal caregiving? These are very different experiences.” (E08)

Terms requiring technical knowledge also posed challenges to comprehensibility. For instance, “trophic features” in Item 23 were unfamiliar to several experts, prompting suggestions for definitions or plain-language alternatives.

Concerns also extended to calibration and response options. Experts questioned whether gradations such as “sometimes” or “a little” were sufficiently anchored to ensure consistent responses. One expert noted:

“Without examples, these words are too subjective—one person’s ‘a little’ could be another’s ‘sometimes.’” (E11)

In the domain of relevance, no items were deemed entirely irrelevant, but several were described as only partially applicable in specific contexts. For example, autonomy-related items were endorsed as important but interpreted differently depending on cultural expectations of independence and role identity:

“For some cultures, family makes decisions collectively, so independence does not mean the same thing.” (E07)

No issues were identified for Perspective Modifiers (0/37), Calibration Across Items (0/37), or Inadequate Response Definition (0/37). Overall, clinicians emphasized clinical applicability and patient comprehension, while doctoral trainees focused more on conceptual clarity and research alignment. This complementary input strengthened the content validation process by addressing both practical and theoretical dimensions of participation.

#### Patient perspectives

Patients flagged 15 items (41%) as problematic, with 10 overlapping with expert concerns and five unique to patient perspectives. Most issues fell under the COSMIN domain of comprehensibility, where unclear or abstract wording led to inconsistent interpretation. For example, Item 4 (“I have difficulty returning to work”) was meaningful for employed participants but not applicable to those retired or engaged in unpaid roles. One participant explained:

“I never had a job outside the home, so this question does not make sense for me. Maybe add examples like housework or caregiving.” (P07, 62-year-old female)

Item 22 (“I avoid participating in social gatherings”) was also interpreted differently. Some patients associated avoidance with physical limitations or pain, while others linked it to stigma and body image:

“It is not that I avoid people—I just do not want them to see my hand like this. That is different from not wanting to go.” (P04, 39-year-old male)

Technical terminology further limited comprehensibility. For instance, Item 23 (“trophic features”) confused several patients:

“What kind of changes do you mean? Skin colour? Temperature? This needs explanation. I had no idea what that word meant.” (P12)

In the domain of relevance, a small number of items were judged only partially applicable. Employment-related questions, for example, did not capture meaningful roles for homemakers or retirees, leading to suggestions for broader contextual examples.

Some concerns are also related to the calibration of responses. Patients noted that vague options such as “sometimes” or “a little” were open to individual interpretation without anchoring examples, raising doubts about response consistency.

No issues were identified for Perspective Modifiers (0/37), Calibration Across Items (0/37), or Inadequate Response Definition (0/37). Patients often attempted to compensate for unclear wording by asking for clarification during interviews or adapting items to their own situations. However, these strategies risked introducing variability in how responses were recorded.

#### Summary of identified issues and modifications

A total of 25 items were flagged through cognitive interviews. Of these, 22 items (88%) underwent revisions to address comprehensibility, four items (16%) were modified to correct reference-point inconsistencies, and 1 item (4%) was adjusted for relevance. No issues were identified for Perspective Modifiers, Calibration Across Items, or Inadequate Response Definition. In several cases, items belonged to more than one category and thus required multiple types of edits. All proposed revisions were reviewed in team meetings and recorded in a decision log. The log showed that all proposals judged necessary for clarity, cultural fit, or applicability were accepted, while no proposals were outright rejected. A small number of suggestions (*n* = 3; e.g., adding extended response options) were deferred to future psychometric testing because they required empirical validation beyond the scope of cognitive interviewing. Overall, the process resulted in revision of 24 out of 37 items (65%), with the remaining 13 items retained in their original wording after team confirmation.

Comprehensibility issues were the most frequent concern, raised in 22 of 37 items (88%) by both experts and patients. These problems reflected unclear wording, technical terminology, or context-dependent phrasing that led to inconsistent interpretation. For example, Item 3 (“I can participate in family activities”) was interpreted variably: some respondents thought of formal gatherings such as holiday dinners, while others associated it with daily caregiving routines. Item 6 (“I cannot use public transportation”) also generated disagreement, as several participants highlighted that their difficulty stemmed not from functional ability but from lack of service availability in rural settings. Terms such as “voluntary job” proved especially problematic, with experts debating whether it referred to community volunteering or informal caregiving, and patients questioning its relevance to their own situations.

Technical language created further barriers. The phrase “trophic features” in Item 23 was not understood by most patients and even some experts, with several requesting definitions or suggesting plainer alternatives such as “changes in skin, colour, or temperature.” Similarly, autonomy-related items were considered important but interpreted differently depending on cultural expectations of independence, with some viewing autonomy in terms of physical self-care and others in terms of decision-making or social independence.

To resolve these concerns, items were revised by replacing technical or ambiguous terms with plain language and by supplementing context-dependent questions with brief examples (e.g., adding “such as household responsibilities” to employment-related items). This ensured that participants across different cultural, social, and clinical contexts could interpret the items consistently without sacrificing the conceptual depth of the construct.

Reference point inconsistencies were identified in four items and raised by nine participants (five experts and four patients). These arose when respondents differed in whether they answered based on their *current abilities*, *past functioning before injury*, or *anticipated abilities after recovery*. For example, Item 4 (“I have difficulty returning to work”) was sometimes interpreted in relation to past employment or expected future return rather than present capacity, particularly among retired participants. Item 10 (“I can manage household responsibilities”) showed similar variation, with some participants describing what they used to do before their condition, while others focused on what they anticipated regaining later. For Item 15 (“I can participate in recreational activities”), experts noted that responses often reflected anticipated post-recovery engagement rather than current participation in these activities. Likewise, Item 27 (“I can participate in social gatherings”) elicited answers based on pre-injury patterns or future expectations rather than present ability.

To address these inconsistencies, the research team revised the anchors to explicitly reference current ability within the past week. Each of the four items was rephrased accordingly (e.g., “In the past week, I have been able to…”), ensuring that all participants interpreted and responded to items consistently in relation to their present functioning. This change standardizes interpretation across respondents and reduces ambiguity between past, current, and anticipated roles.

All flagged items were reviewed by the research team through a structured consensus process. Revisions targeted three main areas. First, comprehensibility issues were addressed by simplifying technical terminology (e.g., replacing *“trophic features”* with plain descriptions of skin changes) and by adding contextual examples to ambiguous phrases such as *“voluntary job”* or *“family roles.”* Second, reference-point inconsistencies were resolved by standardizing anchors to current ability within the past week, ensuring consistency across items that had previously been answered in relation to past or anticipated functioning. Third, relevance concerns were addressed by broadening item examples to include non-employment roles (e.g., caregiving, household responsibilities), ensuring that items applied across diverse life contexts.

All decisions were documented in a log to ensure transparency, and no revisions were made without group consensus. These modifications improved the clarity, cultural sensitivity, and clinical applicability of the English-adapted PBQ, as detailed in Tables 2 and 3.

**Table 2.** Identified issues, original items, and proposed changes for the Participation Behavior Questionnaire (PBQ).

Item	Original text	Issue identified	Feedback source	Participant quote	Proposed change
1	My social activities are reduced (participation in group work etc.).	Vague wording	Patients	“What kind of social activities?”	My social activities, like participating in group work, are reduced.
2	I cannot participate in public places	Misinterpretation	Patients	“What do you mean by public places?”	I cannot participate in public areas, such as parks or shopping centers.
3	I cannot communicate with my colleagues or co-workers	Clear	—	—	No change
4	I cannot get or keep a paid or voluntary job.	Irrelevant	Patients	“This doesn’t apply to my condition.”	I cannot get or keep a job, whether paid or voluntary, that fits my condition.
5	I cannot help others.	Vague wording	Experts	“What do you mean by helping others?”	I cannot help others with tasks or support.
6	I cannot use public transportations.	Misinterpretation	Patients	“Public transport is too broad; examples needed.”	I cannot use public transportation, like buses or trains.
7	I cannot communicate with my friends like before.	Clear	—	—	No change
8	I cannot entertain my relatives and friends in my home.	Vague wording	Experts	“What kind of entertainment?”	I cannot entertain my relatives and friends at home, like hosting gatherings.
9	I mostly try to communicate indirectly (by phone, email, etc.) with others.	Clear	—	—	No change
10	I cannot use public transportations.	Misinterpretation	Patients	“Does public transportation include taxis?”	I cannot use public transportation, including buses and trains.
11	I cannot fulfill my role at home.	Clear	—	—	No change
12	I cannot look after my family	Vague wording	Experts	“Role at home needs more explanation.”	I cannot fulfill my responsibilities in looking after my family.
13	I feel I have lost my autonomy	Misinterpretation	Patients	“Autonomy is too broad.”	I feel I have lost my independence in daily tasks.
14	I cannot take care of myself	Clear	—	—	No change
15	I cannot handle my house works.	Clear	—	—	No change
16	I can no longer look after my home.	Clear	—	—	No change
17	I do not have mastery in doing my daily routines outside of home	Vague wording	Experts	“Daily routines need examples.”	I do not have mastery in doing my daily routines outside of home, such as shopping or errands.
18	I cannot do my self-care independently	Misinterpretation	Patients	“Self-care is too broad.”	I cannot perform self-care tasks independently, like bathing and dressing.
19	I have difficulty in moving around	Clear	—	—	No change
20	I cannot cope with my functional problem.	Clear	—	—	No change
21	My family members are avoiding me.	Clear	—	—	No change
22	The people’s desire to communicate with me has been decreased.	Clear	—	—	No change
23	It is difficult for me to tolerate the reaction of others to the appearance of my hand.	Vague wording	Experts	“Reaction of others needs more context.”	It is difficult for me to tolerate others’ reactions to the appearance of my hand.
24	I feel incompetent like I can no longer participate the way I used to	Clear	—	—	No change
25	I feel dependent on others for doing many of my tasks.	Clear	—	—	No change
26	I feel uncomfortable that my behavior and movements seem inconsistent compared to others.	Clear	—	—	No change
27	I have fewer sport activities than before.	Vague wording	Experts	“Sport activities need examples.”	I have fewer sports activities than before, such as playing tennis or swimming.
28	I cannot participate in entertainment and recreational activities.	Clear	—	—	No change
29	My religious activities have been diminished.	Clear	—	—	No change
30	My entertainment and amusement activities have been limited.	Vague wording	Experts	“Leisure time activities need examples.”	My entertainment and leisure activities, like watching movies or going out, have been limited.

**Table 3.** Proposed changes based on feedback.

Item	Original text	Proposed change
1	My social activities are reduced	My social activities, like participating in group work, are reduced
2	I cannot participate in public places	I cannot participate in public areas, such as parks or shopping centers
4	I cannot get or keep a paid or voluntary job	I cannot get or keep a job, whether paid or voluntary, that fits my condition
5	I cannot help others	I cannot help others with tasks or support
6	I cannot use public transportations	I cannot use public transportation, like buses or trains
8	I cannot entertain my relatives and friends in my home	I cannot entertain my relatives and friends at home, like hosting gatherings
10	I cannot use public transportations	I cannot use public transportation, including buses and trains
12	I cannot look after my family	I cannot fulfill my responsibilities in looking after my family
13	I feel I have lost my autonomy	I feel I have lost my independence in daily tasks
17	I do not have mastery in doing my daily routines outside of home	I do not have mastery in doing my daily routines outside of home, such as shopping or errands
18	I cannot do my self-care independently	I cannot perform self-care tasks independently, like bathing and dressing
23	It is difficult for me to tolerate the reaction of others to the appearance of my hand	It is difficult for me to tolerate others’ reactions to the appearance of my hand
27	I have fewer sport activities than before	I have fewer sports activities than before, such as playing tennis or swimming
30	My entertainment and amusement activities have been limited	My entertainment and leisure activities, like watching movies or going out, have been limited

## Discussion

This study translated and culturally adapted the Participation Behaviour Questionnaire into English (PBQ-E) and evaluated its content validity for individuals with upper extremity conditions in Canada. Cognitive interviews with patients and clinicians identified interpretation challenges related to clarity, contextual relevance, and terminology, guiding targeted revisions to improve comprehensibility and conceptual fidelity. Cognitive interviews with patients and therapists highlighted recurrent challenges related to interpreting items on social participation, autonomy, environmental access, and role engagement, guiding targeted revisions to improve item precision and relevance.^40^ The PBQ-E, as adapted in this pre-psychometric, single-center Canadian English sample, was designed for use by rehabilitation professionals. While our findings support its validity in this context, the applicability of this approach beyond Canadian English rehabilitation remains to be tested, and further research is needed to examine its equivalence across other English-speaking settings. These refinements resulted in more precise wording, improved contextual examples, and better alignment with occupational therapy constructs and the ICF, strengthening the PBQ-E’s ability to capture participation-level challenges in rehabilitation.

Generic participation instruments, such as WHODAS and CIQ, were developed to operationalize participation within the ICF framework; however, ICF-linking studies demonstrate that their coverage of participation domains is incomplete and varies across instruments.^41^ Similarly, disease-specific measures, such as the DASH and PRWE, include items related to work or social participation.^2,42^ However, they are primarily oriented toward impairment and activity limitations, leaving key areas of participation underrepresented. In contrast, the PBQ was explicitly developed from both ICF and occupational therapy perspectives and validated as a four-domain structure, comprising social participation and interpersonal relationships, autonomy and roles, leisure and recreation, and subjective satisfaction with participation.^31^ This broader scope positions the PBQ-E as a complementary tool that captures participation dimensions underrepresented in generic and disease-specific measures, thereby offering added value for assessing participation in upper-extremity conditions.

Cognitive interviewing confirmed that careful cross-cultural adaptation was necessary despite the PBQ’s conceptual clarity. Interpretive challenges—particularly ambiguity, cultural expectations, and reference points—highlighted the importance of pre-testing prior to psychometric evaluation. This aligns with international recommendations emphasizing iterative item refinement to establish content validity before measurement testing.

Cognitive interviewing helped identify item-level interpretation barriers. For example, terms describing social participation (e.g., “public places”) were unclear and needed additional examples; autonomy-related items were often interpreted as decision-making rather than functional ability; and transportation-related wording needed greater specificity to reflect local context. Leisure and recreation items required fewer revisions. These findings echo previous literature emphasizing the importance of pre-testing in establishing cross-cultural and conceptual validity during translation processes.^43,44^ Failure to ensure content validity at this stage can compromise subsequent psychometric evaluations such as reliability or construct validity by introducing measurement errors linked to unclear or culturally incongruent items.

We prioritized clarity and cultural relevance in item wording while remaining attentive to diverse perspectives. Notably, constructs such as autonomy and role participation were interpreted differently across age, gender, and occupational roles, underscoring the need to account for contextual diversity in cross-cultural adaptation. Our approach aligns with international recommendations advocating iterative item refinement based on patient and expert feedback rather than relying solely on linguistic equivalence.^24,45^

One of the most significant findings was that items related to social participation and interpersonal relationships required more refinement to ensure participants accurately interpreted their meaning. The original item, “My social activities are reduced (participation in group work, etc.),” was found to be too vague, with participants questioning the definition of social activities. One patient asked, “What kind of social activities?” leading to a refinement that specified examples such as participating in group work. Similarly, “I cannot participate in public places” was frequently misinterpreted, with a participant questioning, “What do you mean by public places?” To address this ambiguity, the item was revised to “I cannot participate in public areas, such as parks or shopping centers,” explicitly considering environmental and contextual influences on participation.^46^

Another area that required refinement involved autonomy and role performance, where several items were interpreted inconsistently. In particular, participants tended to equate “autonomy” with decision-making rather than functional independence, suggesting that the original wording lacked conceptual clarity. Revisions were therefore made to reflect independence in daily tasks, improving alignment with occupational therapy models such as the Canadian Model of Occupational Performance and Engagement (CMOP-E).^47^

These adaptations strengthened the conceptual precision of the PBQ-E and ensured that the construct of autonomy more accurately reflected lived experience in rehabilitation contexts. Transportation and environmental accessibility also required clarification. Participants noted that access to “public transportation” varies geographically and may encompass multiple modes of travel. Refining wording and adding examples improved the consistency of interpretation across users. These revisions support prior literature showing that environmental factors influencing participation differ across cultures and service infrastructures.^48^

In contrast, leisure, recreation, and sports-related items required minimal modification, suggesting that these constructs may be more universally understood across cultural contexts. Minor changes were made to incorporate examples and enhance clarity, particularly regarding common leisure activities, without altering underlying intent.^49^ Collectively, these patterns illustrate that domains with greater context dependence, such as autonomy/roles and environmental access, require more extensive modification during cross-cultural adaptation, whereas leisure activities appear comparatively stable. This reinforces the value of cognitive interviewing in identifying conceptually vulnerable domains across sociocultural settings.

### Strengths and limitations

A key strength of this study lies in its rigorous and systematic application of established guidelines for the translation and cultural adaptation of the PBQ. The inclusion of both clinical experts and patients ensured a diversity of perspectives, enhancing clinical relevance, linguistic clarity, and conceptual fidelity critical components in cross-cultural adaptation.

Several limitations must be acknowledged. First, recruitment was restricted to a single Canadian tertiary care center, which may limit generalizability; future multi-center studies are needed to capture broader clinical contexts. Second, the expert panel included a substantial proportion of PhD trainees. However, all were licensed clinicians and contributed valuable research-oriented insights; this composition may have affected the balance of perspectives, and future work should prioritize greater representation of practicing clinicians. Third, although subgroup and item-level analyses were reported, formal saturation metrics were not established, and some quantification was limited. Fourth, potential mode effects (in-person vs remote interviews) were not evaluated, and these may have influenced how feedback was elicited. Fifth, Canadian English was the target variant, and adaptations may not generalize to other English-speaking regions with differing cultural and clinical norms. Finally, this pre-psychometric phase did not address downstream psychometric properties such as structural validity, test–retest reliability, responsiveness, or differential item functioning. Scoring implications, including handling of “not applicable” responses, skip logic, and consistent anchoring, also require further evaluation before the PBQ-E can be implemented in clinical or research settings.

## Conclusion

This study translated and culturally adapted the Participation Behaviour Questionnaire (PBQ) into English for individuals with upper extremity conditions and established its content validity through cognitive interviewing with patients and experts. The PBQ-E demonstrates conceptual clarity and cultural relevance within this pre-psychometric, single-center Canadian English context. Notably, the tool addresses domains often overlooked by existing measures—including autonomy, social relationships, leisure, and subjective satisfaction—that both patients and clinicians identified as meaningful for rehabilitation. While these findings suggest that the PBQ-E could enhance clinical assessment of participation, it is not yet ready for routine use. Further research is required to establish the full psychometric properties of the PBQ-E, including structural validity, reliability, responsiveness, and scoring procedures, before it can be confidently implemented in clinical practice.

### Key messages

1. The PBQ-E underwent rigorous translation and cultural adaptation, enhancing its linguistic clarity and conceptual alignment for English-speaking individuals with hand injuries.
2. Cognitive interviews with patients and clinicians refined item content, ensuring the PBQ-E reflects participation constructs aligned with occupational therapy principles and the ICF framework.
3. Content validity has been strengthened, but further psychometric testing (reliability, validity, and responsiveness) is needed before the PBQ-E can be fully implemented in clinical practice.

## Supplemental Material

Supplemental Material - Cross-cultural adaptation and cognitive interview-based content validation of the English Participation Behaviour Questionnaire (PBQ), to measure participation level for individuals with hand injuries Supplemental Material for Cross-cultural adaptation and cognitive interview-based content validation of the English Participation Behaviour Questionnaire (PBQ), to measure participation level for individuals with hand injuries by Maryam Farzad, Rochelle Furtado and Joy MacDermid in Hand Therapy

Supplemental Material - Cross-cultural adaptation and cognitive interview-based content validation of the English Participation Behaviour Questionnaire (PBQ), to measure participation level for individuals with hand injuries Supplemental Material for Cross-cultural adaptation and cognitive interview-based content validation of the English Participation Behaviour Questionnaire (PBQ), to measure participation level for individuals with hand injuries by Maryam Farzad, Rochelle Furtado and Joy MacDermid in Hand Therapy
