# Supplementary material for: Cross-cultural adaptation and cognitive interview-based content validation of the English Participation Behaviour Questionnaire (PBQ), to measure participation in individuals with hand injuries
Source: Hand Ther. 2025 Nov 26:17589983251403595. Online ahead of print. doi: 10.1177/17589983251403595 (PMC12657202; doi:10.1177/17589983251403595)
Supplement: Supplemental Material - Cross-cultural adaptation and cognitive interview-based content validation of the English Participation Behaviour Questionnaire (PBQ), to measure participation level for individuals with hand injuries [file sj-pdf-1-hth-10.1177_17589983251403595.pdf]

PARTICIPATION BEHAVIOUR QUESTIONAIRE

The questions below will help us understand your injury/health condition has affected your participating in important aspects of your life. Please read each statement and out a put a mark X) to indicate if you disagree or agree. 0

Study ID.: …………………. Specified Injury: ………………… Study Visit. …………………

Participation Questionnaire					
**Social Participation and interpersonal relationship**					
		Strongly disagree	Disagree	Agree	Strongly agree
1	My social activities are reduced (participation in group work, etc.).	0	1	2	3
2	I cannot participate in public places				3
3	I cannot communicate with my colleagues or co-workers				3
4	I can not get or keep a paid or voluntary job.				3
5	I cannot help others.				3
6	I can no longer visit with my relatives.				3
7	I cannot communicate with my friends like before.				3
8	I cannot entertain my relatives and friends in my home.				3
9	I mostly try to communicate indirectly (by phone, email, etc.) with others.				3
10	I cannot use public transportations.				
**Autonomy, and role**					
1	I cannot fulfill my role at home.				3
2	I cannot look after my family				3
3	I feel I have lost my autonomy				3
4	I cannot take care of my self				3
5	I cannot handle my house works.				3
6	I can no longer look after my home.				3
7	I do not have mastery in doing my daily routines outside of home				3
8	I cannot do my self-care independently				3
9	I have difficulty in moving around				3
10	I cannot cope with my functional problem.				3
Subjective satisfaction with participation					
1	My family members are avoiding me.				3
2	The people’s desire to communicate with me has been decreased.				3
3	It is difficult for me to tolerate the reaction of others to the appearance of my hand.				3
4	I feel incompetent like I can no longer participate the way I used to				3
5	I feel dependent on others for doing many of my tasks.				3
6	I feel uncomfortable that my behaviour and movements seem inconsistent compared to others.				3
Recreational, sport, and leisure time					
1	I have fewer sport activities than before.				3
2	I cannot participate in entertainment and recreational activities.				3
3	My religious activities have been diminished.				3
4	My entertainment and amusement activities have been limited.				3
