# Supplementary material for: Cross-cultural adaptation and cognitive interview-based content validation of the English Participation Behaviour Questionnaire (PBQ), to measure participation in individuals with hand injuries
Source: Hand Ther. 2025 Nov 26:17589983251403595. Online ahead of print. doi: 10.1177/17589983251403595 (PMC12657202; doi:10.1177/17589983251403595)
Supplement: Supplemental Material - Cross-cultural adaptation and cognitive interview-based content validation of the English Participation Behaviour Questionnaire (PBQ), to measure participation level for individuals with hand injuries [file sj-pdf-2-hth-10.1177_17589983251403595.pdf]

Interview Guide for Cognitive Interviews – PBQ

Purpose:

This guide is designed to assess the clarity, cultural relevance, and conceptual equivalence of the Participation Behaviour Questionnaire (PBQ) in the Canadian context. The questions are structured based on the International Classification of Functioning, Disability, and Health (ICF) and Canadian occupational therapy models (Promoting Occupational Participation [POP] and the Canadian Model of Occupational Participation [CanMOP]) to ensure alignment with participation-based occupational therapy assessments.

Additionally, tailored open-ended questions and specific probes were used to explore participants' reasoning behind their responses. Questions such as "Can you define this word?" or "Can you give an example?" helped assess thought processes and interpretation. Participants also explained the rationale behind their answers. These cognitive interview sessions were recorded and transcribed for analysis by two researchers. Recruitment ceased when response saturation was achieved, defined as three consecutive interviews yielding no new information.

Interview Guide for Patients

Section 1: Demographic Information

1. Age: _______
2. Gender: _______
3. Occupation (if applicable): _______
4. Nature of hand/upper limb injury: _______
5. Time since injury: _______

Section 2: General Understanding of Participation

1. What does **participation** mean to you in your daily life?
2. How has your **injury affected your ability** to participate in meaningful activities?
3. What are the **main barriers** and **facilitators** that influence your participation?

Section 3: Cognitive Interview on PBQ Items

(Questions will be repeated for each PBQ item)

1. **Clarity & Comprehension:**
    - How do you understand this question?
    - Are any words or phrases unclear?
    - Would you suggest alternative wording?
2. **Cultural Relevance:**
    - Does this question reflect **real-life experiences in Canada**?
    - Are there terms that seem inappropriate or unfamiliar?
    - Can you suggest more relevant examples?

3. **Conceptual Equivalence:**
   - Does this question accurately capture your experience of participation?
   - Are there aspects of participation that are missing or need emphasis?

Section 4: Overall Feedback on PBQ Adaptation

1. Do you feel that the PBQ accurately reflects **your participation challenges**?
2. Are there any items that you would **remove, add, or modify**?
3. Does the PBQ provide a complete picture of participation for individuals with hand injuries?
4. Would you feel comfortable using this questionnaire in rehabilitation assessments?

Interview Guide for Therapists

Section 1: Professional Background

1. Years of practice: _______
2. Primary area of practice: _______
3. Experience with participation-based assessments? (Yes/No)
4. Experience working with individuals with hand/upper limb conditions? (Yes/No)

Section 2: Conceptualization of Participation in Practice

1. How do you define **participation** in occupational therapy?
2. What are the most common **participation barriers** faced by your patients with hand injuries?
3. What factors (physical, psychological, environmental, or social) influence participation the most?

Section 3: Cognitive Interview on PBQ Items

(Questions will be repeated for each PBQ item)

1. **Clarity & Comprehension:**
   - How do you interpret this question?
   - Would this wording be **clear and meaningful** for your patients?
   - Are there alternative ways to phrase it?
2. **Cultural & Clinical Relevance:**
   - Does this question align with how **you assess participation** in practice?
   - Does it capture the key challenges that patients experience?
   - Would you modify anything to improve clinical applicability?
3. **Conceptual Equivalence:**
   - Does this question align with **ICF participation domains**?
   - Does it reflect the principles of **POP and CanMOP models**?
   - Are any essential aspects of participation missing?

Section 4: Overall Feedback on PBQ Adaptation

1. Does the adapted PBQ align with **occupational therapy practice in Canada**?
2. Are there items you would **remove, add, or modify**?

3. Would you use this instrument in **clinical practice or research**?
4. Does the PBQ capture participation in a way that is **therapeutically meaningful**?
